# The Notch Pathway: A Link Between COVID-19 Pathophysiology and Its Cardiovascular Complications

**DOI:** 10.3389/fcvm.2021.681948

**Published:** 2021-05-26

**Authors:** Randa M. Breikaa, Brenda Lilly

**Affiliations:** ^1^Center for Cardiovascular Research and The Heart Center, Nationwide Children's Hospital, Columbus, OH, United States; ^2^Molecular, Cellular and Developmental Biology Program, The Ohio State University, Columbus, OH, United States; ^3^Department of Pediatrics, The Ohio State University, Columbus, OH, United States

**Keywords:** notch signaling pathway, COVID-19, cardiovascular, vascular biology, cardiovascular disease

## Abstract

COVID-19 is associated with a large number of cardiovascular sequelae, including dysrhythmias, myocardial injury, myocarditis and thrombosis. The Notch pathway is one likely culprit leading to these complications due to its direct role in viral entry, inflammation and coagulation processes, all shown to be key parts of COVID-19 pathogenesis. This review highlights links between the pathophysiology of SARS-CoV2 and the Notch signaling pathway that serve as primary drivers of the cardiovascular complications seen in COVID-19 patients.

## Introduction

Beginning in December 2019, the world faced a challenging nemesis presented by a member of the coronaviruses family, SARS-CoV2, later known as Coronavirus Disease 2019 or COVID-19 (1–3). First feared for its aggressive attack on the respiratory system (4, 5), it is now recognized for its severe cardiovascular complications (6–8). These range from hemodynamic instabilities, dysrhythmias, and thromboembolic events, to myocarditis, acute heart failure and cardiac arrest (9, 10). Analyzing patient data from several countries, cardiovascular disease appears in two contexts associated with COVID-19. First, studies have shown that pre-existing cardiovascular disease increases the risk of COVID-19 infection and is indeed present in a high number of cases (11–13). Second, COVID-19 patients develop cardiovascular complications during the course of the disease (14, 15). Despite a clear connection with COVID-19 and the cardiovascular system, we understand little about this relationship.

Notch signaling is a master regulator of cardiovascular function in both health and disease, and has been linked to several biological processes mediating viral infections (16, 17). A recent study by Rosa et al., characterized transcriptional signatures induced in a rhesus macaque model of SARS-CoV2 and showed an increase in Notch signaling in the lungs of the macaques (18). Another group studying human protein interactions with SARS-CoV2 using computational models, showed that proteins interacting with the 5'-region of SARS-CoV2 RNA were associated with Notch2 receptor signaling (19). The Notch pathway is also implicated in the hypoxic response and in coagulopathic processes, both of which are present in COVID-19 patients. These known roles of the Notch pathway make this signaling pathway a likely player in the COVID-19-driven cardiovascular complications.

## The Beginning (Viral Entry)

The angiotensin converting enzyme 2 (ACE2) has been established to play a significant role in SARS-CoV viruses infectivity, including COVID-19, by binding to the viral spike protein and facilitating entry into the host cell (20, 21). ACE2 has distinct roles in the body, ranging from amino acid transportation and catalytic activities, to serving as functional receptors for viruses like the coronaviruses. In the heart, it is localized to cardiomyocytes, cardiac fibroblasts, epicardial adipose tissue, and the coronary vascular endothelium. In the lungs, it is expressed on the cell surface of the inner respiratory tract, protecting against lung injury. This protective effect stems from its negative regulation of the renin-angiotensin system which leads to the inhibition of the vasoconstrictive, pro-inflammatory angiotensin II (ANGII)—ANGII type 1 receptor (AT1) axis (22–24). Its unique location in both organs combined with its function make it a pivotal player in the pulmonary pathogenicity of the virus and its associated cardiovascular complications. Thus, ACE2 on one hand offers protection against injury, while on the other hand facilitates viral entry. Furthermore, upon binding of ACE2 to the viral particle, the receptor itself becomes endocytosed by the cells causing depletion of cell surface ACE2 and its mediated tissue protection (25, 26). This dilemma and the realization of the importance of ACE2 in maintaining cardiovascular homeostasis drove attempts to manipulate the ACE2/ANGII axis to mitigate virus-induced injury, while minimizing the negative effects on the protective functions of ACE2 (20, 23). One solution for this problem and an attractive target for vaccine development are the viral S-proteins, which when targeted make the enzyme unable to bind, preventing viral entry (21, 27).

Notch signaling has been known to interact with many viral particles facilitating their infectivity (Table 1). Given that Notch regulates various proliferative and differentiation events in cells, it is no surprise that the pathway is an attractive target for viruses, which are dependent on the cell cycle machinery of the cell. Those viruses tap into the Notch pathway to ensure their own survival (60–62). The first evidence that demonstrated Notch pathway-viral interactions was reported for the Epstein-Barr virus, which targets RBPJ (mouse)/CBF1 (human), the nuclear effector of Notch (28, 63). Other examples include the human papilloma virus (HPV), hepatitis B virus (HBV), and hepatitis C virus (HCV). In the case of HCV, the Notch1 receptor has been shown to facilitate nuclear localization of p65 in response to tumor necrosis factor-alpha (TNF-α) in human hepatocytes, leading to increased pathogenicity of the virus (64). Additionally, the influenza virus has been shown to block the Notch ligand Delta-like 1 (DLL1) causing a heightened inflammatory response and decreased interferon-c levels, which leads to compromised immunity against the virus. In contrast, macrophages were found to enhance their DLL1 production during the course of infection to protect against the same virus (32, 33, 65). In the case of COVID-19, an interesting enzyme that could be linking Notch and COVID-19 activation is FURIN. FURIN is a member of the protein convertases family and is both an activator and a direct target of Notch activity (66, 67). Its enzymatic activity has been proven to be exploited by a variety of bacteria and viruses, including measles, yellow fever, ebola, and avian influenza, thereby facilitating their virulence and spread (68, 69). To discern the potential role of FURIN in COVID-19, understanding the structure of the viral S-glycoprotein is important. The S-protein has two functional domains: one for receptor binding and the other for mediating fusion of the viral particle with the cell membrane. The S-protein must be cleaved by the protease to expose these fusion sequences and allow cell entry. FURIN takes on this role in coronaviruses including COVID-19 (70–72). Since Notch1 has been shown to transcriptionally induce FURIN, Notch signaling may indirectly lead to enhanced viral entry via enhanced FURIN expression (73, 74).

**Table 1:** Reported link of the Notch signaling pathway to common viral infections.

**Viral infection**	**Link to notch**	**References**
Epstein-barr virus	The Epstein-Barr virus nuclear antigen 2 (EBNA2) is tethered to promoters by targeting RBPJ, the nuclear effector of Notch. Since EBNA2 has been proven to be partly interchangeable with Notch intracellular domain in activation of target genes modulating differentiation processes, it is seen as a biological equivalent of an activated Notch receptor. The Epstein-Barr virus-encoded latent membrane protein 2A (LMP2A) promotes cellular migration mediated by Notch signaling by altering mitochondrial dynamics.	(28–31)
Influenza virus	Macrophages are reported to enhance their Notch ligand DLL1 production in response to the viral infection to protect against the virus. Blocking DLL1 caused heightened inflammatory response and decreased interferon-c levels, leading to a compromised immunity against the virus.	(32–34)
Respiratory syncytial virus (RSV)	Notch signaling has been reported to contribute to the production of inflammatory cytokines induced by the virus in alveolar macrophages. Notch signaling communicates with the Toll-like receptor (TLR) pathway to fine-tune the innate inflammatory responses. In studies where TLR pathway was activated, while Notch signaling was inhibited, RSV-enhanced respiratory disease (ERD) was prevented.	(35, 36)
Human papilloma virus (HPV)	Notch inhibition impairs epithelial differentiation, which is suggested to contribute to HPV replication and viral oncogenesis. HPV8E6 protein inhibits Notch transcriptional activator complexes involving RBPJ and MAML at the Notch target genes, decreasing Notch activity during keratinocyte differentiation. HPV16E6 protein increases Notch levels in keratinocytes. HPV16E6 potentiates Notch activation and differentiation without activating cellular arrest, entirely uncoupling cellular arrest from increased differentiation.	(37–42)
Human T-cell leukemia virus type 1 (HTLV-I)	Notch signaling promotes proliferation and tumor formation of HTLV-I-associated adult T-cell leukemia.	(43, 44)
Hepatitis C virus (HCV)	Notch signaling regulates T Helper 22 Cells in chronic HCV patients. Notch1 receptor has been shown to facilitate nuclear localization of p65 in human hepatocytes in response to TNF-α, leading to increased pathogenicity of the virus. HCV NS3 protein leads to Notch activation by binding to SRCAP transcription factor. HCV causes Notch-dependent modulation in miRNA-449a levels, leading to differential expression of the inflammatory biomarker YKL40.	(45–48)
Hepatitis B virus (HBV)	HBV increases Notch1 and TGF-β levels on intrahepatic T cells in cirrhosis, promoting fibrogenesis and disease progression. HBV X protein activates Notch signaling by increasing DLL4 and Notch1, promoting the growth of hepatocellular carcinoma, in addition to increasing CREB-mediated activation of miR-3188. HBV X protein causes Notch-dependent decrease in nuclear factor-kappa B (NF-κB) signaling. Notch signaling contributes to hepatic inflammation in HBV infection by regulating IL-22-producing cells. Notch signaling aids in transcription of HBV covalently closed circular DNA by a mechanism involving cAMP response element-binding protein and E3 ubiquitin ligase-modulation. In acute hepatitis B (AVH-B) infection, a complementary association between Notch1 and Hes1 in CD8^+^T cells was reported. In chronic hepatitis B (CHB) infection, repression of the Notch receptors mediates the immune response regulation in patients who progress to cirrhosis and hepatocellular carcinoma.	(49–55)
Human immunodeficiency virus (HIV)	Notch signaling is activated in HIV-associated nephropathy, where Notch ligands (Jagged-1, Jagged-2, DLL1, and DLL4) are all up in kidney tubules, while glomeruli show minimal ligand expression. Notch1 and 4 receptors are up in glomeruli, and only Notch4 is expressed in tubules. Notch inhibition results in improvement of kidney injury scores and renal functions, and blocks podocyte proliferation induced by HIV proteins Nef and Tat.	(56–59)

In addition to having effects on viral infectivity, interestingly both the Notch receptors and ACE2 receptor share a common mechanism of activation through cleavage by the A disintegrin and metalloproteinase (ADAM) family of enzymes, specifically ADAM17 (75, 76). ADAM17 mediates ectodomain shedding of ACE2 which can facilitate viral entry (77, 78). ADAM17 also activates the Notch signaling pathway via receptor cleavage leading to increased viral infectivity through regulation of FURIN. Therefore, Notch activity is indirectly involved in COVID-19 infectivity through FURIN induction and shared activation axis of ACE2, both of which aid in viral entry.

## The Cytokine Storm

A balanced innate and adaptive host immunity is key for an effective antiviral response, including activation of T cells, macrophages, and production of various pro-inflammatory cytokines. However, in case of COVID-19, this response becomes heightened, causing a hyperinflammatory reaction known as “The Cytokine Storm Syndrome” (14, 27). The cytokine storm is one of the key factors causing cardiovascular complications in COVID-19 patients. This is attributed to the resulting inflammation-induced vascular injury, myocarditis, arrhythmia, and destabilization of coronary artery plaques leading to myocardial infarcts (79, 80). The common profile of a COVID-19 patient with cytokine storm syndrome includes elevated interleukin-6 (IL-6), IL-2 receptor, TNF-α, granulocyte-colony stimulating factor, among others. IL-6 is secreted by activated leukocytes, promotes differentiation of B lymphocytes and production of acute phase proteins, and is important for thermoregulation (14, 81).

The role of the Notch pathway in inflammation is well-documented, where it has been shown to promote the pro-inflammatory microenvironment (82–84). It is implicated in macrophage polarization and contributes to amplification of the inflammatory loop by promoting the M1 phenotype of macrophages over the M2 phenotype (17, 85). Furthermore, in macrophages, Notch1 directly binds the IL-6 promoter and activates IL-6 transcription in response to interferon-γ (81, 86). Additionally, IL-6 in turn increases the expression of the Notch ligand DLL1, amplifying the Notch signal. This works as a positive feedback loop that further drives the production of more IL-6 (87, 88). Nitric Oxide Synthase (iNOS) expression is linked to manifestation of the cytokine storm (89, 90). Direct interaction between the Notch Intracellular Domain (NICD) and TNF-α on the iNOS promoter has also been documented, indicating multiple avenues by which Notch signaling drives hyper-inflammation (91). Further, TNF-α itself has been shown to induce expression of Notch1 and Notch4, in addition to regulating NICD nuclear translocation, which leads to the activation of Notch downstream mediators (92, 93).

This interplay between Notch and pro-inflammatory processes makes the Notch pathway an attractive target for reversing inflammatory events. Indeed, genetic and pharmacological inhibition of Notch signaling was reported to ameliorate disease progression in many inflammatory disease models. These include rheumatoid arthritis, autoimmune encephalomyelitis, and several models of infectious disease (94, 95). In the case of COVID-19, the recommendation to use corticosteroids was discouraged due to controversial efficacy and reports showing exacerbation of patient symptoms. Potentially targeting the Notch pathway to specifically block the inflammatory loop re-enforced by IL-6 and TNF-α may present a viable therapy for these cases (96–98).

## The Hypoxic Response

The hypoxic events in COVID-19 patients have been a mystery to medical caretakers and physicians. This is due to the fact that the patients display minimal visible distress, although clinical oxygen levels are remarkably low (99). Its presentation defies its pathophysiology, which initially led to its description of “Happy Hypoxia” (100). Hypoxia is also linked to the thrombotic events seen in these patients, which spirals quickly into more severe cardiovascular complications such as myocarditis and myocardial infarction (101–103).

The Notch pathway plays a significant role in hypoxic events (104). Notch3 is induced under hypoxic conditions in the lungs and vasculature. Notch3 deletion has been shown to protect against the development of pulmonary arterial hypertension in response to hypoxic stimulation (105, 106). Further, Notch3 was found to cooperate with the hypoxia-inducible factor-1 alpha (HIF-1α) (105, 107), a transcription factor upregulated in hypoxia and inflammatory microenvironments and a master regulator of oxygen homeostasis (108). HIF-1α also induces the expression of two of the Notch ligands, DLL4 and Jagged-1 (109–111). Another link between Notch signaling and HIF-1α is through Notch1 receptor. As mentioned previously, Notch1 receptor has been shown to promote M1 macrophage polarization and switching of macrophage metabolism to glycolysis. This is followed by induction of M1 gene transcription, coupled with an increase in mitochondrial oxidative phosphorylation and generation of reactive oxygen species (112). This in turn activates HIF-1α to induce M1 macrophage activation, in a type of positive feedback loop (111).

Additionally, enhanced Notch signaling has been linked to structural changes in air sacs in the lungs that include decreased septation of terminal alveoli, emphysematous patterns and progressive fibrotic changes (113). Furthermore, Notch3 plays a critical role in regulating alveolar epithelium and increased levels of Notch3 are associated with disruption of differentiation processes and altered lung morphology (114). Interestingly in COVID-19-associated hypoxia, the air sacs do not fill up with fluid like in pneumonia, but also show structural changes in the sacs that lead them to collapse (115, 116). Hence, Notch activation in COVID-19 patients is likely directly exacerbating the hypoxic events by cooperating with HIF-1α in addition to promoting structural defects in the air sacs.

## The Coagulopathic Response

The realization that COVID-19 causes hypercoagulopathy poses more questions than answers, with studies showing severe thrombotic manifestations, while others show postmortem lung sections with extensive bleeding (117, 118). In a recent study by Boonyawa et al. a 28% incidence of venous thromboembolism was reported in COVID-19 patients in the intensive care unit (119). Another study by Klok et al. found a 31% incidence of combined deep vein thrombosis, pulmonary embolism, and arterial thrombosis in critically ill patients (120). Thus, there is an urgent need to understand the rate of bleeding and thrombotic events associated with COVID-19 coagulopathy.

Hypercoagulopathy is an important hallmark of inflammation. In fact, pro-inflammatory cytokines are directly involved in accelerating platelet hyperactivation and driving thrombotic events, while impairing crucial physiological anticoagulation pathways including antithrombin III, tissue factor pathway, and the protein C system (121, 122). The mechanisms involved in COVID-19 coagulopathy have not been fully elucidated yet, but crosstalk between the coagulation and the inflammatory systems is evident, with at least four factors seeming to contribute to this condition (123). First, the pro-inflammatory mediators such as IL-6 and IL-1β produced during the cytokine storm stimulate the production of tissue factor on immune cells. This in turn initiates the activation of the extrinsic coagulation cascade (81, 124). Secondly, those pro-inflammatory mediators directly activate the platelets themselves (125). Thirdly, a decrease in plasminogen activator coupled with an increase in plasminogen activator inhibitor suppresses the fibrinolytic system (126, 127). Lastly, the damage caused to the endothelial cells by the inflammatory reaction results in vascular homeostatic imbalances, causing accelerated local thrombotic events in addition to systemic coagulation defects. Of note is that this damaged endothelium also binds platelets more readily due to enhanced platelet-vessel wall interaction caused by the large von Willebrand factor multimers released by damaged endothelial cells (128).

Despite efforts by the scientific community to understand COVID-19-associated coagulopathy, there is still a lot to clarify regarding mechanisms involved and how to reverse the resulting homeostatic imbalances. Previous studies by Duarte et al. and Gough beautifully demonstrated a link between the Notch pathway and the coagulation pathway through fibroblast growth factor 1 (FGF1) (129, 130). These studies utilized a soluble form of the Notch ligand Jagged-1 to show the effect of Notch inhibition on FGF1 and the coagulation cascade. This link between Notch signaling and coagulation is supported by several previous findings. First, the activation of the coagulation cascade by damaged tissue generates thrombin, which activates the protease-activated receptor 1 (PAR1) and PAR1-dependent FGF1 expression and release. Released FGF1 subsequently promotes angiogenesis and induces Jagged-1 expression in the damaged tissue (131). Second, Alagille syndrome patients, who primarily have mutations in Jagged-1, show bleeding disorders (132). Consistent with this, Jagged-1 null mice show hemorrhage during their embryonic development (133, 134). Lastly, Jagged-1 was found to be the FGF1 response gene responsible for FGF1-dependent endothelial cell differentiation on fibrin matrices (131, 135). Taken together, these studies indicate that there is a Notch-dependent mechanism by which thrombin can regulate FGF1 secretion, which in turn contributes to thrombin's activity, the key protease of the coagulation cascade. Thus, through its established role in both inflammation and coagulation, Notch signaling seems likely responsible for exacerbating COVID-19-associated coagulopathy.

## Endothelial Cell Involvement

The endothelium is a single layer of cells lining blood vessels, constituting a barrier between the circulation and the rest of the blood vessel wall. In addition, it is the source for several vasoreactive substances responsible for blood vessel contraction and relaxation such as endothelin and nitric oxide (136). Thus, it is a key regulator of vascular homeostasis, and damage to this layer can lead to loss of the homeostatic state and exacerbation of disease conditions. Indeed, endothelial dysfunction shifts the vascular equilibrium toward an inflammatory, pro-coagulant state (137, 138). Coordination of leukocyte trafficking in particular is critical for the inflammatory response. Under physiological conditions, the endothelial cells prevent binding and extravasation of leukocytes from the blood. However, under disease conditions such as the case in COVID-19 patients, the endothelial junctions are weakened and leaky, resulting in facilitated exit of the leukocytes from the circulation into the tissues (82, 93, 139). Interestingly, immunostaining studies have shown the confinement of Notch4 receptors on endothelial cells at the apical membrane. This localization makes Notch4 ideal for receptor/ligand communication between the endothelial and the inflammatory cells in the blood stream (140). In addition, the Notch ligand DLL4 on the endothelium has been shown to trigger a bidirectional Notch signaling between endothelial cells and monocytes (93, 141).

Several reports have linked endothelial cells to SARS-CoV2 pathology, where histological sections through hearts, kidneys and lungs showed accumulation of both inflammatory cells and viral particles within the endothelium (81, 142, 143). This COVID-19-associated endotheliitis could explain the impaired circulatory function in the various vascular beds and the clinical complications in COVID-19 patients (144–146). Furthermore, endothelial cells express the ACE2 receptor, the entry portal for the virus (147–150). This, coupled with previous reports of development of autoantibodies against endothelial cells after SARS-CoV1 infection, suggests that CoV2 infection of endothelial cells and their subsequent damage is a prominent step in the pathogenesis of COVID-19 (151, 152). Important to consider here is the discrepancy that exists between current studies, where some advocate for the endothelial cell hypothesis of COVID-19 pathology, which reinforces the idea that endothelial cells are the origin for COVID-19-associated cardiovascular impairments. In contrast, others promote a pericyte-COVID-19 hypothesis, where pericytes are the main contributors to disease progression. The studies that propose the pericyte hypothesis are based on the fact that ACE2 expression in the heart is highest in pericytes, and in the brain vasculature ACE2 is on vascular smooth muscle cells and pericytes and not on the endothelium (121, 153). Although the endothelial cell hypothesis seems to be more plausible according to consequences of endotheliitis in COVID-19 patients, these discrepancies highlight the importance of considering tissue type in disease pathology.

## Conclusions

**Figure 1:**
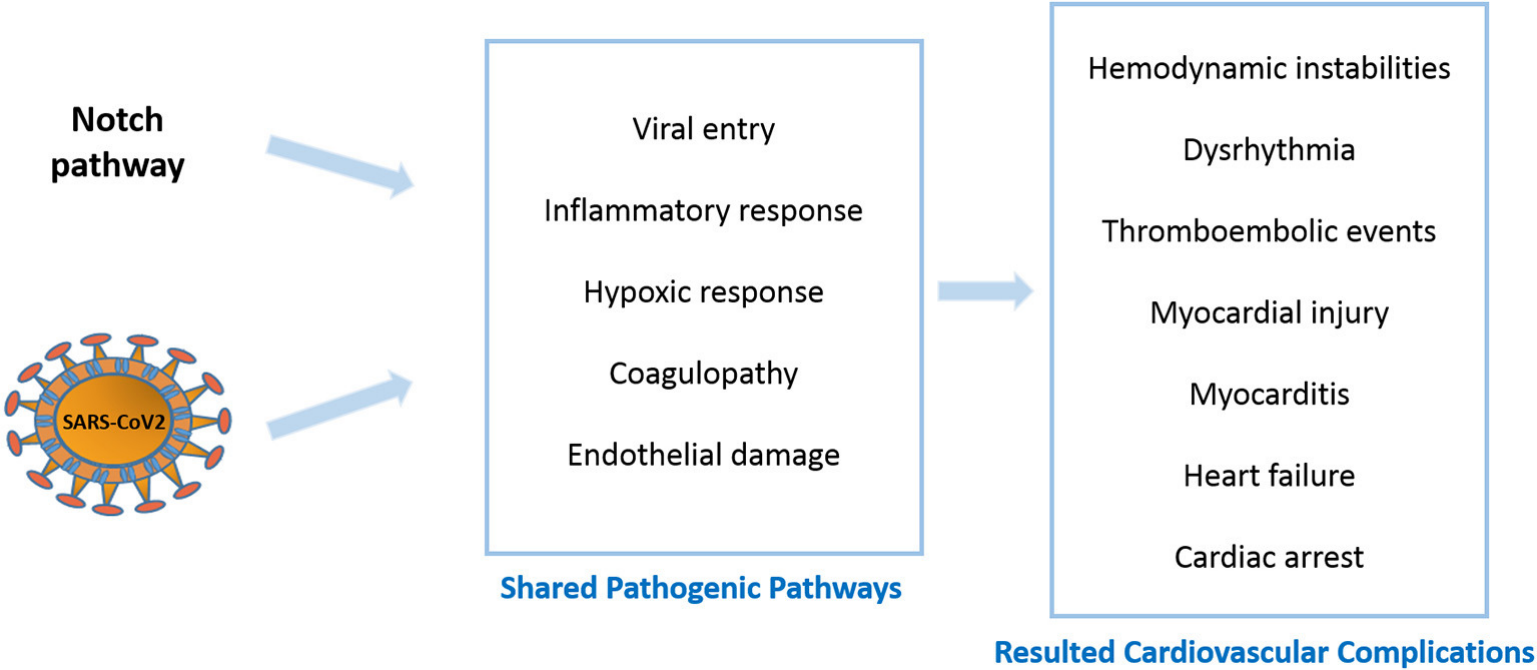
Notch signaling and COVID-19 shared pathogenic events and resulted cardiovascular complications.

COVID-19 is associated with a large number of cardiovascular sequelae, including dysrhythmias, myocardial injury, myocarditis and thrombosis. Many of these complications seem to be linked to compromised signaling pathways in the patients, including the Notch pathway (Figure 1). Notch signaling can indirectly enhance viral entry through inducing FURIN, the protease responsible for exposing the fusion sequences of the viral S-protein. The established role of Notch signaling in both inflammation and coagulation suggests its involvement in COVID-19 cytokine storm and hypercoagulopathy, both of which are main contributors to the cardiovascular complications. Furthermore, Notch activation is known to exacerbate hypoxic events by cooperating with HIF-1α in addition to enhancing structural defects in the air sacs in the lungs, which together may contribute to enhanced lung pathology in COVID-19 patients. Lastly, the suggested role of the endothelium in COVID-19 cardiovascular impairments coupled with the specific localization of Notch4 receptors on the apical membrane of the endothelium reinforces the idea that the Notch pathway serves as a communication channel between endothelial and inflammatory cells.

In summary, several scenarios can be considered regarding the link between the Notch pathway and COVID-19-associated cardiovascular events. COVID-19 may act upstream to increase Notch signaling, leading to enhanced viral entry and associated pathogenic processes. Alternatively, maladaptive responses of Notch signaling due to COVID-19 infection may contribute to the enhanced inflammatory, coagulopathic, and hypoxic events. Both scenarios eventually lead to exacerbation of cardiovascular impairments in COVID-19 patients that are Notch-associated. Gamma-secretase inhibitors, which inhibit Notch receptor cleavage have been used to attenuate Notch signaling in cancer and Alzheimer disease (154, 155). These compounds, however, are associated with significant toxicity. Alternatives include Notch-specific antibodies and decoys. Antibodies allow blockade of individual Notch components, thus are not associated with complications seen with the pan inhibitors (156–158). Notch decoys also selectively block Notch receptors by a unique mechanism that involves mimicking the Notch extracellular domain of a specific Notch ligand or receptor (159, 160). Finally, uncovering new aspects of a Notch-COVID-19 relationship might help mitigate cardiac and pulmonary complications caused by the SARS-CoV family of viruses.

## Author Contributions

RB wrote, edited, and conceived of topic. BL edited, wrote, and provided topic guidance. All authors contributed to the article and approved the submitted version.

## Conflict of Interest

The authors declare that the research was conducted in the absence of any commercial or financial relationships that could be construed as a potential conflict of interest.
